# Macrophage Sphingosine 1-Phosphate Receptor 2 Blockade Attenuates Liver Inflammation and Fibrogenesis Triggered by NLRP3 Inflammasome

**DOI:** 10.3389/fimmu.2020.01149

**Published:** 2020-06-26

**Authors:** Lei Hou, Le Yang, Na Chang, Xinhao Zhao, Xuan Zhou, Chengbin Dong, Fuquan Liu, Lin Yang, Liying Li

**Affiliations:** ^1^Department of Cell Biology, Municipal Laboratory for Liver Protection and Regulation of Regeneration, Capital Medical University, Beijing, China; ^2^Department of Interventional Therapy, Beijing Shijitan Hospital, Capital Medical University, Beijing, China

**Keywords:** macrophage, NLR family pyrin domain-containing 3, inflammation, sphingosine 1-phosphate, S1P receptor 2, liver injury

## Abstract

NLR family pyrin domain containing 3 (NLRP3) inflammasome accompanies chronic liver injury and is a critical mediator of inflammation-driven liver fibrosis. Sphingosine 1-phosphate (S1P)/S1P Receptor (S1PR) signaling participates in liver fibrogenesis by affecting bone marrow (BM)-derived monocytes/macrophage (BMM) activation. However, the relationship between S1P/S1PR signaling and NLRP3 inflammasome in BMMs remains unclear. Here, we found significantly elevated gene expression of NLRP3 inflammasome components (NLRP3, pro-interleukin-1β, and pro-interleukin-18) and the activation of NLRP3 inflammasome significantly elevated during murine chronic liver injury induced by a bile duct ligation operation, a methionine-choline–deficient and high-fat diet, or carbon tetrachloride intraperitoneal injection. Moreover, the increased expression of sphingosine kinase 1 (SphK1), the rate-limiting synthetic enzyme of S1P, was positively correlated with NLRP3 inflammasome components in both patients and mouse model livers. Flow cytometry analysis and immunofluorescence staining showed BMMs contributed to the significant proportion of NLRP3^+^ cells in murine inflammatory livers, but not Kupffer cells, dendritic cells, endothelial cells, T cells, and hepatocytes. Focusing on macrophages, S1P promoted NLRP3 inflammasome priming and activation in a dose-dependent manner. Blockade of S1PR_2_ by JTE-013 (antagonist of S1PR_2_) or S1PR_2_-siRNA inhibited S1P-induced NLRP3 inflammasome priming and inflammatory cytokine (interleukin-1β and interleukin-18) secretion, whereas blockade of S1PR_1_ or S1PR_3_ had no such effect. *in vivo*, a β1,3-d-glucan-encapsulated siRNA particle (GeRP) delivery system is capable of silencing genes in macrophages specifically. Treatment with S1PR_2_ siRNA-GeRPs markedly reduced NLRP3 inflammasome priming and activation and attenuated liver inflammation and fibrosis. Together, the conclusions indicated that targeting macrophage S1PR_2_ retarded liver inflammation and fibrogenesis via downregulating NLRP3 inflammasome, which may represent an effective therapeutic strategy for chronic liver injury.

## Introduction

The inflammasomes are oligomeric complexes formed by innate immune sensors, including NOD-like receptor (NLR) family members NLRP1b, NLRP3, and NLRC4, as well as other non-NLR receptors, such as AIM2 (1). Recent results have shown that the expression of hepatic NLRP3 inflammasome components increases during liver fibrogenesis (2), and NLRP3 expression level is closely correlated with the severity of liver fibrosis (3). Aberrant activation of NLRP3 inflammasome results in severe liver inflammation with immune cell infiltration, hepatic stellate cell activation, and collagen deposition (4, 5). Classical activation of the NLRP3 inflammasome requires two independent steps (6). The first step has been referred to as priming, in which activators, such as lipopolysaccharide (LPS), induce nuclear factor-κB (NF-κB) and mitogen-activated protein kinase (MAPK)-dependent expression of NLRP3 inflammasome components, including NLRP3 and pro-interleukin (IL)-1β (6). The second step has been referred to as activation. Upon detection of an activation signal, the adapter molecule ASC links the NLRP3 protein to the recruited caspase-1, resulting in the activation of caspase-1 (6). Active caspase-1 p10/20 cleaves its target substrates pro-IL-1β and pro-IL-18 into their released mature forms (6).

Sphingosine 1-phosphate (S1P) has emerged as bioactive molecules generated from sphingosine by sphingosine kinase 1 (SphK1) and SphK2 (7, 8). It has been reported that S1P can directly act as intracellular signaling molecules or function as the natural ligand of five different types of S1P receptors (S1PRs) (7–9). Recently, S1P/S1PRs signaling has emerged as a crucial regulator of various inflammatory-related diseases, such as atherosclerosis, rheumatoid arthritis, and multiple sclerosis, as well as cholestasis-induced liver injury (10). In patient and mouse model injured livers, S1P levels and the expression of SphK1 and S1PRs are markedly upregulated, whereas the mRNA expression of SphK2, S1P phosphatases, and S1P lyase show little changes (11–13). Overactive SphK1/S1P/S1PRs signaling exerts powerful pro-inflammatory and pro-fibrotic effects, and thus aggravates tissue damage (11, 12, 14–20). Our previous studies showed that S1P mediates the angiogenic process in hepatic stellate cells required for fibrosis development during liver injury via S1PR_1_ and S1PR_3_, whereas S1P/S1PR_2_ signaling is involved in promoting hepatic stellate cell contraction (12, 21, 22). S1P/S1PR_2/3_ signaling plays a significant role in liver injury by affecting the infiltration and pro-inflammatory M1 polarization of bone marrow-derived monocyte/macrophages (BMMs) (14–16). In addition, the substantial secreted inflammatory cytokines caused by S1P/S1PR signaling directly participate in chronic inflammation and tissue damage, such as IL-6 and tumor necrosis factor (TNF)-α (14–16, 19). Furthermore, blockade of S1P/S1PR_2_ signaling significantly reduces secretion of inflammatory cytokines, enhances regenerative response of hepatocytes, and ameliorates the inflammation and fibrosis during liver injury effectively (14–18, 23, 24). These effects suggest that targeting S1P/S1PRs signaling will be viewed as a promising strategy for the treatment of liver fibrosis. Owing to S1PRs are widely expressed in various kinds of cells, traditional delivery methods-systemic administration can lead to undesirable systemic side effects (25–28). Thus, efficiently delivering pharmacological inhibitors or siRNA into specific cells is expected to be a more effective therapy for the treatment of liver diseases.

Here, we displayed that NLRP3 mainly expressed and functioned in BMMs via an overactive S1P/S1PR_2_ system, highlighting the key role of macrophage S1PR_2_ as an effective therapeutic target of NLRP3 inflammasome-dependent sterile inflammation. Previous results have shown that a β 1,3-d-glucan-encapsulated siRNA particle (GeRPs) delivery system is capable of silencing genes in macrophages specifically (29, 30). In our study, selective knockdown of macrophage S1PR_2_ by S1PR_2_ siRNA-GeRPs downregulated NLRP3 inflammasome and retarded liver fibrogenesis, which may represent a novel therapeutic strategy for chronic liver injury.

## Materials and Methods

### Reagents

Sphingosine 1-phosphate (Cay62570-1, S1P) and D-erythro-Dihydrosphingosine 1-phosphate (AG-CR1-0005, H_2_S1P) were from Biomol (Tebu, France). Lipopolysaccharide (L2630, LPS) was from Sigma-Aldrich (St. Louis, MO). ATP (NLRP3 inflammasome inducer) was from Invitrogen (tlrl-atpl, Grand Island, NY, USA). MCC950 (NLRP3 inflammasome inhibitor) was from MedChemExpress (HY-12815, Monmouth Junction, NJ, USA). W146 (10009109, S1PR_1_ antagonist), JTE-013(10009458, S1PR_2_ antagonist), and CAY-10444(10005033, S1PR_3_ antagonist) were from Cayman Chemical (Ann Arbor, MI).

### Animal Models

Male ICR mice, 31.0 ± 1.0 g, at 6 weeks of age were used in this study. Bile duct ligation (BDL) mice were anesthetized and induced by BDL. Sham-operated mice, used as controls, underwent a laparotomy with exposure, but no ligation of the common bile duct was performed. Mice were anesthetized and sacrificed at 1 or 3 days or at 1 or 2 weeks after BDL (*n* = 6 per group). Methionine-choline-deficient and high-fat (MCDHF) mice and carbon tetrachloride (CCl_4_) mice, in detail, mice were fed either a control diet or an MCDHF diet (A06071309, Research Diet) containing 46 kcal% fat, 18 kcal% protein, and 36 kcal% carbohydrate. Then mice were anesthetized and sacrificed at 28 days (*n* = 6 per group). A CCl_4_ (1 μL/g BW)/olive oil (OO) mixture (1:9 v/v) was injected into the abdominal cavity of mice twice per week (*n* = 6 per group). Then mice were anesthetized and sacrificed at 4 weeks. All animal work was conformed to the Ethics Committee of Capital Medical University and were in accordance with the approved guidelines (approval AEEI-2014-131).

### Human Specimen

Human fibrotic samples (fibrosis stage: F2: 3, F2/F3: 10, F3/F4: 9, F4: 1) were obtained from livers of 23 patients undergoing liver biopsy (12 men, 11 women; mean age: 58 year; range: 25–79 year). Fibrosis was caused by chronic HCV (*n* = 4) or HBV (*n* = 8) infection, cholestatic (*n* = 1) or alcoholic (*n* = 3) liver cirrhosis, drug-induced liver injury (*n* = 1), cryptogenic cirrhosis (*n* = 4), and autoimmune liver disease (*n* = 2). Normal liver samples were collected from eight patients undergoing hepatic resection for hepatic hemangioma (3 men, 5 women; mean age: 45 year; range: 29–59 year). All subjects gave their informed consent for inclusion before they participated in the study. The study was conducted in accordance with the Declaration of Helsinki, and the protocol was approved by the Ethics Committee of Beijing Shijitan Hospital, Capital Medical University, Beijing, China (Project identification code: 2018EC-1).

### Immunofluorescence Staining

BMMs were fixed by 4% paraformaldehyde and penetrated by 0.2% Triton X-100 (Amresco, OH, USA). After being blocked with 2% BSA (Roche, Switzerland), they were incubated with rabbit anti-ASC polyclonal antibody (AG-25B-0006, 1:400, Adipogen, San Diego, USA). FITC-conjugate affinipure goat-anti-rabbit IgG (111-095-144, 1:400, Jackson Immunoresearch, PA, USA) was used as a secondary antibody. Nuclei were stained with DAPI.

The liver specimen was fixed in 4% paraformaldehyde, and frozen sections of 5 μm were used for immunofluorescence. Frozen sections were incubated with rat anti-F4/80 polyclonal antibody (sc-71085, 1:400, Santa Cruz Biotechnology, Santa Cruz, CA) and mouse anti-NLRP3/NALP3 monoclonal antibody (AG-20B-0014, 1:400, Adipogen, San Diego, USA) as the first antibody. Cy5-conjugated goat anti-rat IgG (1:400, 112-175-143) and Cy3-conjugated goat anti-mouse IgG (1:400, 115-165-062) was from Jackson ImmunoResearch Laboratories and used as a secondary antibody. Finally, the sections were stained with DAPI and observed under a confocal microscope (LSM510, Carl Zeiss MicroImaging, Jena, Germany).

### Flow Cytometry

Isolation of mouse liver non-parenchymal cells was as described in this section. Subsequently, antibodies including anti-F4/80-APC (17-4801-82), anti-CD11c-APC (17-0114-82), anti-CD3e-APC (17-0031-82), and anti-CD31-APC (17-0311-82, eBioscience, San Diego, CA) were added to the cell suspension, respectively. After 30 min of incubation at 4°C in the dark, the cells were washed with PBS and incubated with fixation/permeabilization buffer (eBioscience, San Diego, CA). Then, the cells were washed and stained with anti-NLRP3/NALP3-750 (IC7578S, R&D Systems, Eugene, Oregon) for 60 min at 4°C and analyzed with a flow cytometer. FACS was performed on a FACSAria and analyzed with FACSDiva 4.1 (BD Biosciences).

### Mouse Primary Liver Macrophage and Hepatocyte Isolation

Livers were minced with scissors and digested at 37°C for 30 min in TESCA buffer containing collagenase (1 mg/mL; Sigma-Aldrich) and DNase I (5 μg/ml; Sigma-Aldrich). After treatment with RBC Lysis Buffer (Gibco; Grand Island, NY), hepatocytes and macrophages were separated using low-speed centrifugation and 70%/30% percoll density gradient centrifugation (middle layer), respectively.

### Preparation and *in vivo* Administration of Glucan-Encapsulated siRNA Particles

Cy3-labeled glucan shells were prepared as previously described (29). To prepare a GeRP, 3 nmol siRNA (Dharmacon) were incubated with 15 nmol Endo-Porter (GT17EPD, EP, Gene Tools) in 30 mM sodium acetate (pH 4.8) for 30 min at room temperature in a final volume of 200 μl. Then, 0.3 mg of Cy3-labeled glucan shells were dissolved in 50 μl 30 mM sodium acetate (pH 4.8) and sonicated to ensure homogeneity of the GeRPs. The siRNA-EP solution was added to GeRPs and then vortexed and incubated for 90 min. Tris/EDTA buffer (10 mM Tris and 1 mM EDTA, pH 8.0) was added to the particles and incubated for 15 min at room temperature to adjust the pH to 7.4. The siRNA-loaded GeRPs were then resuspended in PBS aliquoted into tubes for daily dosing and either flash-frozen in liquid nitrogen or stored at −80°C.

Male ICR mice, 31.0 ± 1.0 g, at 6 weeks of age were anesthetized and induced by bile duct ligation (BDL) or sham operation. The i.p. injection of S1PR_2_ siRNA-GeRPs (1.5 mg GeRPs/kg of body weight) or Nc siRNA-GeRPs (negative control, 1.5 mg GeRPs/kg of body weight) was performed 1 day after operation, then once a day with the same dose for 12 days. Mice were anesthetized and sacrificed at 2 weeks after BDL or sham operation (*n* = 6 per group). Then, the selective uptake of GeRPs in macrophages (F4/80^+^) in BDL-treated livers was measured by confocal microscopy. Approximately 300 macrophages (F4/80^+^) or hepatocytes (large nuclear cells) were measured by the software Image-Pro Plus per simple. The mean value of six randomly selected samples was used as the expressed percentage of Cy3^+^ cells in total macrophages or hepatocytes.

### Histology Analysis

Liver tissue sections (5 μm) were stained with H&E staining for assessment of inflammation and Sirius Red staining for the extent of collagen deposition. The hepatic inflammatory foci or area of fibrosis was measured by computer-assisted image analysis with Leica Qwin V3 software. The mean value of 15 randomly selected areas per sample was used as the expressed percentage of hepatic inflammatory foci and fibrotic area.

### BMM Acquisition

Male ICR mice, 31.0 ± 1.0 g, at six weeks of age were anesthetized and sacrificed by cervical dislocation at the time of bone marrow (BM) harvest. BM cells were extracted from the tibias and femurs by flushing with culture medium. Extracted BM cells were cultured for 7 days in the presence of L929-conditioned medium. BMMs were serum-starved for 6 h and then used to perform the following experiments.

### RNA Interference (RNAi) *in vitro*

siRNA sequences targeting specifically mouse S1PR_1_, S1PR_2_, and S1PR_3_ were from Invitrogen (Thermo Scientific, PA, USA). Forty to fifty percent of confluent BMMs were prepared. Transient transfection of siRNA (40 nmol/L) was used by employing Lipofectamine RNAi MAX (Invitrogen, Carlsbad, CA) as recommended by the manufacturer. Scramble siRNA was used as a negative control. Cells were used to fulfill further experiments after 48 h.

### Western Blot Analysis

Antibodies were as follows: mouse anti-NLRP3/NALP3 monoclonal antibody (AG-20B-0014, 1:2,000, Adipogen, San Diego, USA); rabbit anti-IL-1β monoclonal antibody (#12426, 1:2,000, Cell Signaling Technology, Beverly, MA, USA); rabbit anti-IL-18 polyclonal antibody (ab71495, 1:2,000, Abcam, Cambridge, UK); mouse anti-caspase-1 (p20) monoclonal antibody (AG-20B-0042, 1:2,000, Adipogen, San Diego, USA); and the appropriate IRDyeTM 800-conjugated secondary antibody (926-32210, 926-32211, 1:10,000, li-cor). Results were normalized relative to β-tubulin (HC101-01 1:5,000, Transgen Biotech, China) expression.

### Measurement of IL-1β and IL-18 by ELISA

The concentration of IL-1β and IL-18 was measured by an IL-1β ELISA kit (BMS6002, Invitrogen, Carlsbad, CA) and an IL-18 ELISA kit (CSB-E04609m, CUSABIO, Houston, USA) according to the manufacturer's instructions. A standard curve was created, and results were normalized to the protein content of the sample.

### qRT-PCR

Total RNA was extracted from frozen liver specimens or cultured BMMs, with or without treatments, then qRT-PCR was performed. Primer sequences are listed in Table 1.

**Table 1:** Primer sequence.

**Mouse**	**Sequence**	
18s rRNA	Sense	GTAACCCGTTGAACCCCATT
	Antisense	CCATCCAATCGGTAGTAGCG
sphingosine kinase 1 (Sphk1)	Sense	ATGGAACCAGTAGAATGCCCT
	Antisense	TCCGTTCGGTGAGTATCAGTTTA
NLR family, pyrin domain containing 3 (Nlrp3)	Sense	ATTACCCGCCCGAGAAAGG
	Antisense	TCGCAGCAAAGATCCACACAG
interleukin 1 beta (Il1β)	Sense	GCAACTGTTCCTGAACTCAACT
	Antisense	ATCTTTTGGGGTCCGTCAACT
interleukin 18 (Il18)	Sense	GACTCTTGCGTCAACTTCAAGG
	Antisense	CAGGCTGTCTTTTGTCAACGA
sphingosine-1-phosphate receptor 1 (S1pr1)	Sense	ACTTTGCGAGTGAGCTG
	Antisense	AGTGAGCCTTCAGTTACAGC
sphingosine-1-phosphate receptor 2 (S1pr2)	Sense	TTCTGGAGGGTAACACAGTGGT
	Antisense	ACACCCTTTGTATCAAGTGGCA
sphingosine-1-phosphate receptor 3 (S1pr3)	Sense	TGGTGTGCGGCTGTCTAGTCAA
	Antisense	CACAGCAAGCAGACCTCCAGA
NLR family, CARD domain containing 4 (Nlrc4)	Sense	ATCGTCATCACCGTGTGGAG
	Antisense	GCCAGACTCGCCTTCAATCA
absent in melanoma 2 (Aim2)	Sense	GTCACCAGTTCCTCAGTTGTG
	Antisense	CACCTCCATTGTCCCTGTTTTAT
NLR family, pyrin domain containing 1B (Nlrp1b)	Sense	AGTAATCTGGAGGGGTTGGAC
	Antisense	GTTGGCAGCCAGGGTATATCA
NLR family, CARD domain containing 3 (Nlrc3)	Sense	CAGATTGGTAACAAAGGAGCCA
	Antisense	CGTTCGGTTTATCTTCAGAGCA
NLR family, pyrin domain containing 2 (Nlrp2)	Sense	GAAAGCTGGACAAGACTGAGTT
	Antisense	GGCAGTGGTCTGTGAGAATTT
NLR family, pyrin domain containing 4a (Nlrp4a)	Sense	GGATGCCCAAAGTTATATCGAGC
	Antisense	CCAGGCCAGCATTAACCTCTT
NLR family, pyrin domain containing 5 (Nlrp5)	Sense	GAAAGCACAATGGGTCCTCCA
	Antisense	CTGACGCCTGTTCCACTTCT
NLR family, pyrin domain containing 6 (Nlrp6)	Sense	CTCGCTTGCTAGTGACTACAC
	Antisense	AGTGCAAACAGCGTCTCGTT
NLR family, pyrin domain containing 9a (Nlrp9a)	Sense	CTTTGCTGCAATATCCAAGGGA
	Antisense	GGACAGCGGCATAAATTGAACA
NLR family, pyrin domain containing 10 (Nlrp10)	Sense	TCAAGACGCTGAAGTTCCACT
	Antisense	TGCTCCGTACATTGAAATCAGTT
NLR family, pyrin domain containing 12 (Nlrp12)	Sense	AAGACCGCAATGCACGATTAG
	Antisense	TGGAGCGTTCCCACTCTACA
NLR family, pyrin domain containing 14 (Nlrp14)	Sense	TCCACAAACGGTAGTCCTTCA
	Antisense	ACTTGTCCCTGTTCAATGGGG
NLR family member X1 (Nlrx1)	Sense	TAGGGCCTTTATCCGTTACCA
	Antisense	TAAACCACTCGGTGAGGTTCC
NLR family, apoptosis inhibitory protein 1 (Naip1)	Sense	TGCCCAGTATATCCAAGGCTAT
	Antisense	AGACGCTGTCGTTGCAGTAAG
NLR family, apoptosis inhibitory protein 2 (Naip2)	Sense	AGCTTGGTGTCTGTTCTCTGT
	Antisense	GCGGAAAGTAGCTTTGGTGTAG
NLR family, apoptosis inhibitory protein 5 (Naip5)	Sense	TGCCAAACCTACAAGAGCTGA
	Antisense	CAAGCGTTTAGACTGGGGATG
NLR family, apoptosis inhibitory protein 6 (Naip6)	Sense	TACAGGGAGTTTACAAGACCCC
	Antisense	AGTGGCCTGGAGAGACTCAG
**Human**	**Sequence**	
NLR family, pyrin domain containing 3 (NLRP3)	Sense	GATCTTCGCTGCGATCAACAG
	Antisense	CGTGCATTATCTGAACCCCAC
interleukin 1 beta (IL1β)	Sense	TTCGACACATGGGATAACGAGG
	Antisense	TTTTTGCTGTGAGTCCCGGAG
interleukin 18 (IL18)	Sense	TCTTCATTGACCAAGGAAATCGG
	Antisense	TCCGGGGTGCATTATCTCTAC
sphingosine kinase 1 (SPHK1)	Sense	AGAGTGGGTTCCAAGACACCT
	Antisense	GGGTGCAGCAAACATCTCAC

### Statistical Analysis

Results are expressed as mean ± SEM. Comparisons between two independent groups were performed using a two-sample *t*-test. Comparisons between multiple groups were performed by one-way or two-way ANOVA with *post-hoc* Tukey's multiple comparison tests when appropriate. Correlation coefficients were calculated by Pearson test. *p* < 0.05 was considered significant. All results were verified in at least three independent experiments.

## Results

### Liver Injury of Different Etiologies Was Accompanied by Elevated NLRP3 Inflammasome Priming and Activation

**Figure 1:**
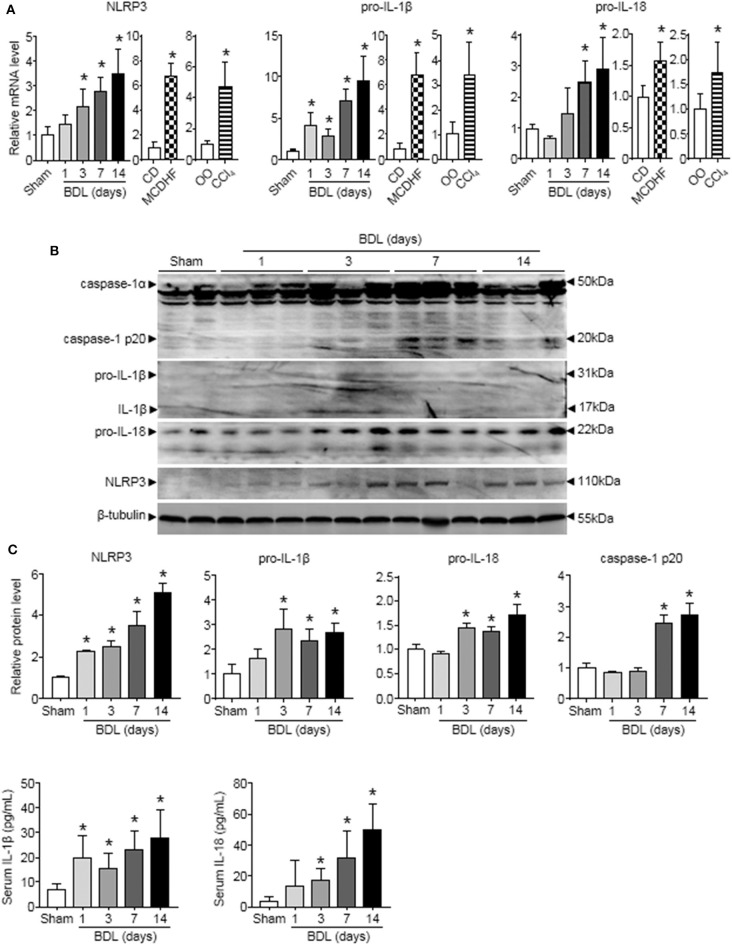
The dynamic changes of NLRP3 inflammasome priming and activation during chronic liver injury. The adult mice received an operation of bile duct ligation (BDL), methionine-choline-deficient and high-fat (MCDHF) diet, or carbon tetrachloride (CCl_4_) treatment to induce liver injury. **(A)** Expressions of NLRP3, pro-IL-1β, and pro-IL-18 in mouse livers were measured by RT-qPCR. **(B)** NLRP3, pro-IL-1β, pro-IL-18, and caspase-1 (cleaved caspase-1 p20 and un-cleaved caspase-1) protein expression from sham-operated and BDL-treated mouse livers were analyzed and quantified by Western blot. **(C)** ELISA analysis of IL-1β and IL-18 levels in serum. Data are presented as the mean ± SEM. *n* = 6 per group. Comparisons between two independent groups were performed using a student's *t*-test. One-way ANOVA was used for panels **(A–C)**. *p < 0.05 vs. control group.

To examine the role of NLRP3 inflammasome in liver injury of different etiologies, we first detected the changes of NLRP3 inflammasome components (NLRP3, pro-IL-1β, and pro-IL-18) expression in three mouse models of liver injury induced by bile duct ligation (BDL), methionine-choline-deficient and high-fat (MCDHF) diet, or carbon tetrachloride (CCl_4_). qRT-PCR analysis showed that NLRP3, pro-IL-1β, and pro-IL-18 mRNA increased continuously throughout the entire stage of BDL-induced liver injury (Figure 1A). Similar results were obtained in liver samples from mice treated with MCDHF diet and CCl_4_ (Figure 1A). Second, Western blot analysis revealed a pronounced increase in NLRP3, pro-IL-1β, and pro-IL-18 expression in BDL-treated livers (Figure 1B). Third, we detected the dynamic changes of NLRP3 inflammasome activation in BDL-treated livers. The protein level of cleaved caspase-1 p20 rose significantly and got to the topmost level after 2 weeks (Figure 1B). Meanwhile, ELISA assay revealed that IL-1β and IL-18 in serum were significantly increased after 3 days of BDL operation (Figure 1C). These results supported the importance of NLRP3 inflammasome in chronic liver injury.

### BMMs Contributed to a Significant Proportion of NLRP3^+^ Cells in Injured Livers

**Figure 2:**
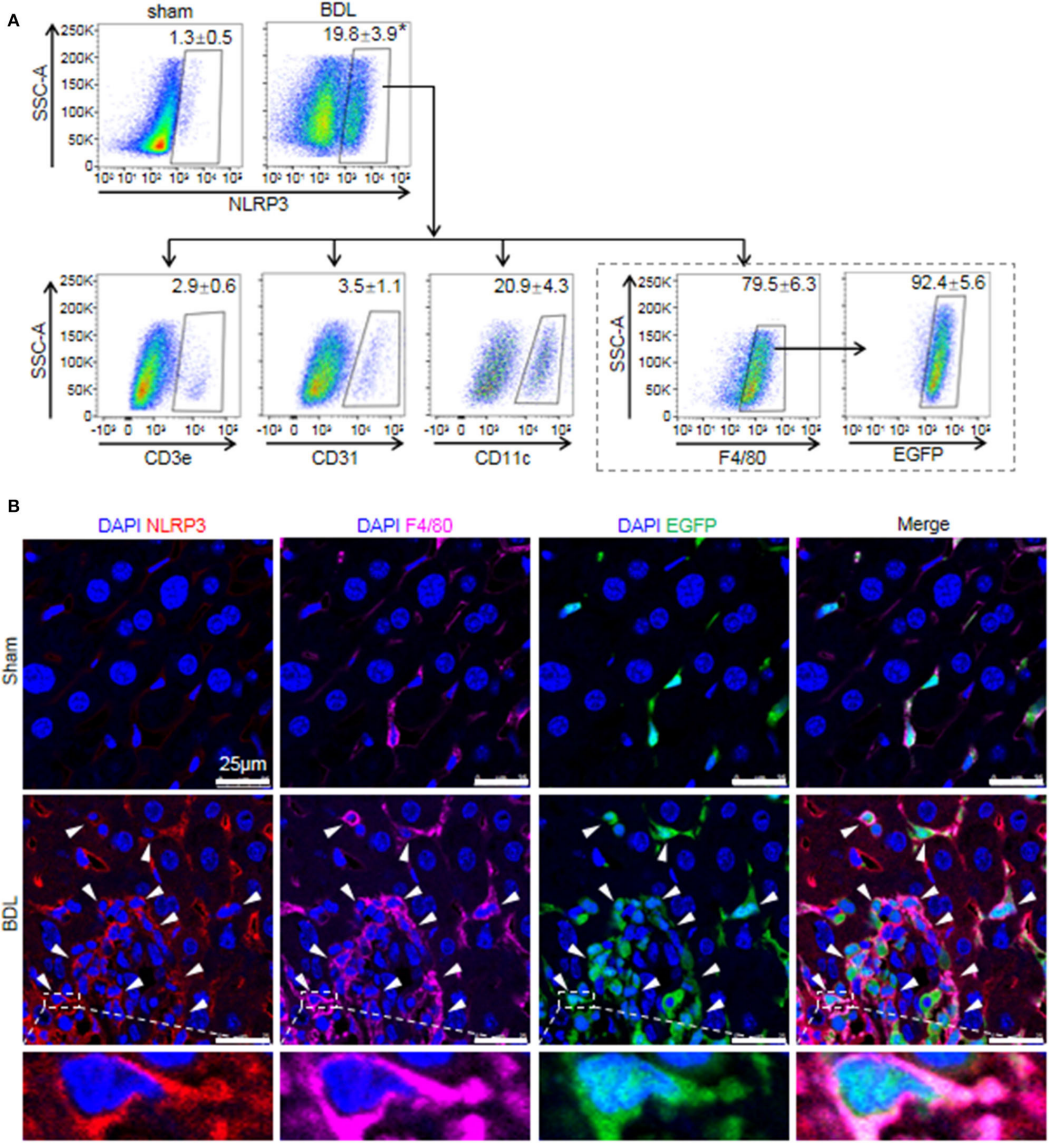
The cell-specific expression pattern of NLRP3 in the fibrotic liver of BDL-treated mice. **(A)** Mice were anesthetized and sacrificed at 2 weeks after sham or BDL operation. NLRP3 positive cells of the mouse liver non-parenchymal cells were analyzed for T cells, endothelial cells, dendritic cells, and macrophages-specific markers (CD3e, CD31, CD11c, and F4/80, respectively). Mice were lethally irradiated and received whole bone marrow cell transplants from EGFP transgenic mice, followed by sham or BDL operation. At 2 weeks after sham or BDL operation, the proportion of NLRP3^+^ cells in BMMs (F4/80^+^ EGFP^+^) was analyzed by FACS. **(B)** Representative images of immunofluorescence analysis by confocal microscopy to track the expression of NLRP3 (red) in macrophages (F4/80^+^, pink) of bone marrow origin (EGFP^+^, green) in mouse liver tissue. Data are presented as the mean ± SEM. *n* = 6 per group. Student's *t*-test was used in panel **(A)**. *p < 0.05 vs. sham group.

While previous studies provided evidence that NLRP3 existed and was functionally active in various hepatic non-parenchymal cells (5, 31), they did not clarify the main origin of NLRP3^+^ cells. Thus, we investigated the cell-specific expression patterns of NLRP3 in mouse hepatic non-parenchymal cells. FACS analysis showed that the proportion of NLRP3^+^ cells in non-parenchymal cells of BDL-treated livers increased markedly compared with that in sham-operated mouse livers (Figure 2A). In addition, the percentage of NLRP3^+^ macrophages (F4/80^+^) accounted for 79.5 ± 6.3% of total NLRP3^+^ cells, whereas dendritic cells (CD11c^+^, 20.9 ± 4.3%), endothelial cells (CD31^+^, 3.5 ± 1.1%) and T cells (CD3e^+^, 2.9 ± 0.6%) showed much lower NLRP3 expression (Figure 2A).

There are two kinds of macrophages in damaged liver: resident Kupffer cells and recruited BMMs (32). Kupffer cells are a population of macrophages resident within the liver, where they locally proliferate and self-sustain (32). To further clarify the expression of NLRP3 in Kupffer cells and BMMs, we performed an EGPF^+^ bone marrow cell transplantation experiment, followed by BDL-induced liver injury. In previous research, we performed F4/80 staining in the chimera mice with BDL or CCl_4_-induced liver injury, and we noted that most of Kupffer cells (F4/80^+^ and EGPF^−^) were alive and not replaced by bone marrow monocytes (33, 34). Here, almost all of NLRP3^+^ macrophages were BMMs (F4/80^+^ and EGFP^**+**^) in BDL-injured livers, supporting the important role of BMMs in NLRP3-driven immune responses (Figure 2A). Consistently, immunofluorescence staining showed that significant numbers of NLRP3^+^ cells were also positive for F4/80 and EGFP in BDL-treated livers, whereas NLRP3 was undetectable in hepatocytes (large nuclear cells) in our study (Figure 2B). Altogether, our data identified that BMMs contributed to a significant proportion of NLRP3^+^ cells and might act as an important part in NLRP3 inflammasome priming and activation during BDL-induced liver injury.

### Macrophage NLRP3 Inflammasome Priming and Activation Was Induced by S1P in Dose-Dependent Manner

**Figure 3:**
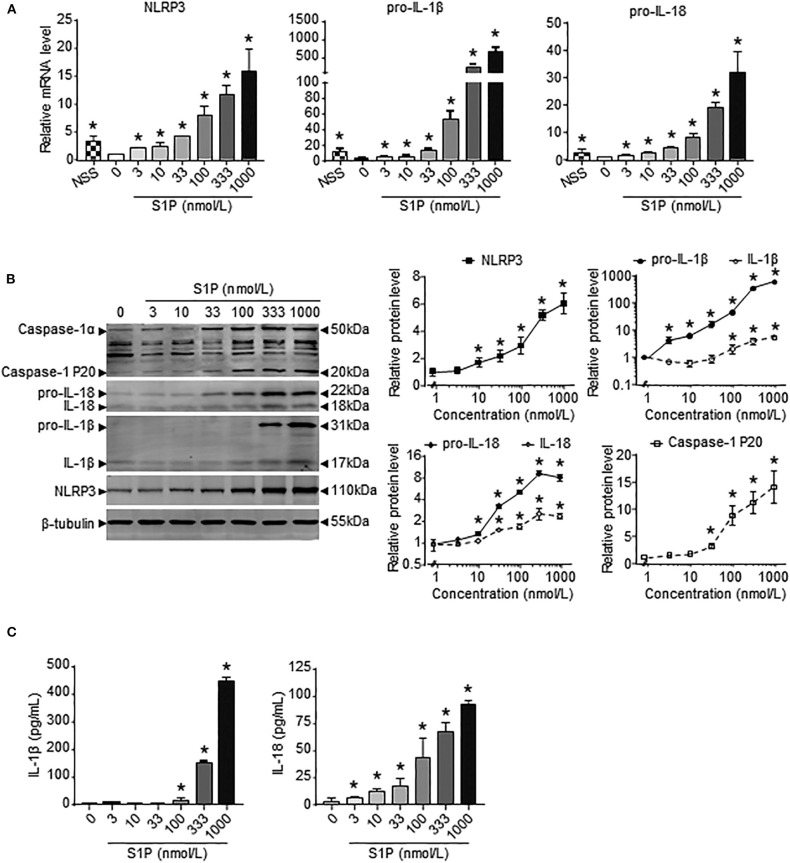
S1P increased NLRP3 inflammasome priming and activation in dose-dependent manner. **(A)** NLRP3, pro-IL-1β, and pro-IL-18 mRNA expression were examined by RT-qPCR in non-serum-starved BMMs (NSS), and serum-starved BMMs treated with the indicated concentrations of S1P for 2 h. **(B)** NLRP3, pro-IL-1β, IL-1β, pro-IL-18, IL-18, and caspase-1 (cleaved caspase-1 p20 and un-cleaved caspase-1) protein expression were examined and quantified by Western blot in serum-starved BMMs treated with S1P for 12 h. **(C)** ELISA measurements of IL-1β and IL-18 in culture supernatants from serum-starved BMMs treated with S1P for 12 h. Data are presented as the mean ± SEM. *n* = 6 per group. One-way ANOVA was used for all comparisons. *p < 0.05 vs. control.

Previous results showed that the upregulation of SphK1 expression resulted in increased S1P concentration in injured liver (11, 12); thus, we speculated whether SphK1/S1P signaling was involved in NLRP3 inflammasome priming and activation. Here, we noted that the mRNA levels of NLRP3 inflammasome components (NLRP3, pro-IL-1β, and pro-IL-18) exerted positive correlations with the expression of SphK1, the rate-limiting synthetic enzyme of S1P, in both human and mouse livers (Table 2). Then, serum-starved BMMs were treated with S1P to validate the expression of NLRP3 inflammasome components. qRT-PCR and Western blot analysis showed that S1P induced a pronounced increase in NLRP3, pro-IL-1β, and pro-IL-18 expression in dose-dependent manner (Figures 3A,B). Bone marrow monocytes differentiated with colony-stimulating factor (M-CSF) are known to prime the macrophage toward the M2 phenotype (35), whereas serum used to culture BMMs contains a large number of nutritional and macromolecular factors essential for cell growth, including S1P (36). Here, we noted that NLRP3, pro-IL-1β, and pro-IL-18 mRNA expression in non-serum-starved BMMs was significantly higher than that in serum-starved BMMs (Figure 3A). We also detected the mRNA expression of M1 markers (TNF-α and IL-6) and M2 markers (Arginase-1, CD206, and CD163) in non-serum-starved BMMs, serum-starved BMMs, and serum-starved BMMs treated with S1P. qRT-PCR analysis revealed that Arginase-1, CD163, and IL-6 mRNA expression in non-serum-starved BMMs were significantly higher than that in serum-starved BMMs, while there was no change of CD206 and TNF-α levels in non-serum-starved and serum-starved BMMs (Supplementary Figure 1). Our data supported that L929-conditioned media (M-CSF) induced the M2 phenotype in BMMs. In addition, S1P induced a pronounced increase in the mRNA expression of M1 markers (TNF-α and IL-6) in serum-starved BMMs, whereas S1P had no effects on M2 marker (Arginase-1, CD206, and CD163) mRNA expression (Supplementary Figure 1). These results validated that S1P treatment promoted pro-inflammatory M1 macrophage polarization in BMMs.

**Table 2:** The correlation between SphK1 and NLRP3, pro-IL-1β, or pro-IL-18 in the liver.

**Parameter**		**NLRP3**		**pro-IL-1β**		**pro-IL-18**	
		***r***	***p*-value**	***R***	***p*-value**	***r***	***p*-value**
SphK1	Human	0.69	<0.05	0.91	<0.05	0.75	<0.05
	Mouse	0.77	<0.05	0.73	<0.05	0.63	<0.05

*Male ICR mice, 31.0 ± 1.0 g, at 6 weeks of age received an operation of BDL (n = 24), MCDHF diet (n = 6), or CCl_4_ treatment (n = 6) to induce liver injury. Sham-operated mice (n = 6), OO-treated mice (n = 6), and mice fed with control diet (n = 6) were used as controls. Human liver samples were obtained from livers of 31 patients with hepatic fibrosis (n = 23) or hepatic hemangioma (n = 8). The mRNA expression of SphK1, NLRP3, pro-IL-1β, and pro-IL-18 in human and mouse livers were quantified using qRT-PCR. Then, the relationship between SphK1 and NLRP3, pro-IL-1β, and pro-IL-18 was analyzed by regression analysis*.

**Figure 4:**
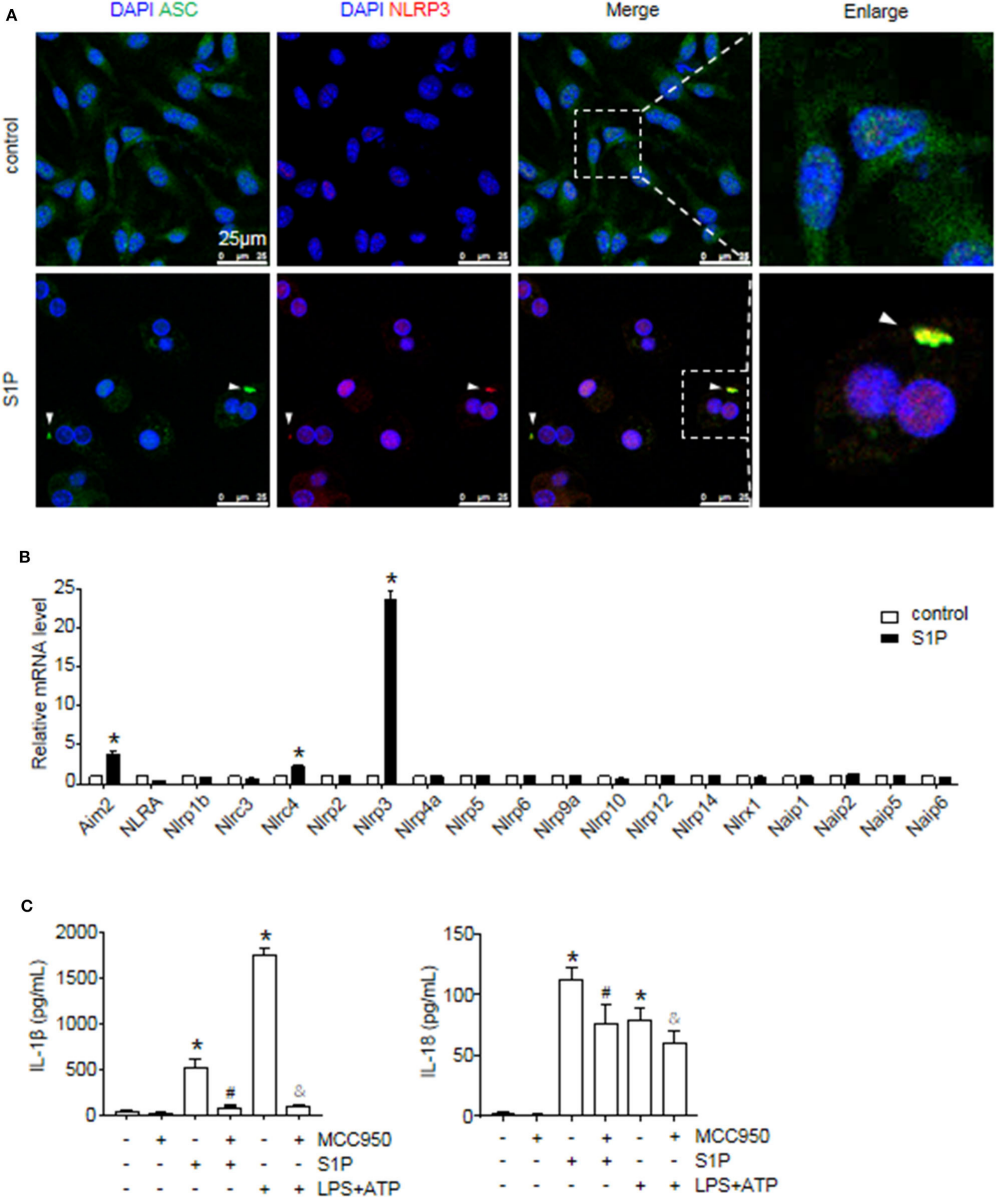
S1P induced IL-1β and IL-18 secretion in NLRP3 inflammasome-dependent manner. **(A)** Representative images of immunofluorescence analysis by confocal microscopy in serum-starved BMMs treated with 1 μmol/L S1P for 6 h. White arrows indicate oligomerized ASC. **(B)** The relative expression of NLR and non-NLR PRR family members were examined by qRT-PCR in serum-starved BMMs treated with S1P for 2 h. **(C)** ELISA measurements of IL-1β and IL-18 in culture supernatants from serum-starved BMMs pretreated with 100 nmol/L MCC950 for 1 h, followed by treatment with 1 μmol/L S1P for 12 h, or 100 ng/ml LPS for 4 h, then 5 mmol/L ATP for 30 min. Data are presented as the mean ± SEM. *n* = 6 per group. Student's *t*-test was used in panel **(B)** and one-way ANOVA was used in panel **(C)**. *p < 0.05 vs. control. ^#^*p* < 0.05 vs. S1P treatment alone. ^&^*p* < 0.05 vs. LPS plus ATP treatment.

Next, we investigated the ability of S1P to stimulate NLRP3 inflammasome activation. Western blot analysis revealed that S1P increased cleaved caspase-1 (caspase-1 p20), mature IL-1β, and IL-18 production in dose-dependent manner (Figure 3B). ELISA assay showed that BMMs secreted significant amounts of IL-1β and IL-18 in response to S1P in dose-dependent fashion (Figure 3C). In addition, we found that ASC was evenly distributed throughout unstimulated BMMs. In contrast, NLRP3 was found in small foci that predominantly colocalized with ASC specks in S1P-treated BMMs, which were indicative of NLRP3 inflammasome activation (Figure 4A).

The macrophage can express other intracellular pattern recognition receptors (PRRs), which are also capable of forming inflammasomes, such as NLRP1b, NLRC4, and AIM2 (1). Thus, we examined whether S1P induced IL-1β and IL-18 secretion dependent on NLRP3 inflammasome. Here, we found that S1P specifically induced NLRP3, AIM2, and NLRC4 mRNA expression, whereas the mRNA fold changes of AIM2 and NLRC4 were much lower than that of NLRP3 (Figure 4B). Then, we pretreated BMMs with MCC950 to further confirm the specificity of S1P-mediated activation of NLRP3 inflammasome (37). Treating BMMs with MCC950 resulted in a strong reduction in the release of mature IL-1β and IL-18 (Figure 4C), indicating that S1P-induced secretion of IL-1β and IL-18 was specifically dependent on NLRP3 inflammasome activation.

### S1P Promoted NLRP3 Inflammasome Priming and Increased IL-1β and IL-18 Secretion via S1PR_2_, but Not S1PR_1_ and S1PR_3_

**Figure 5:**
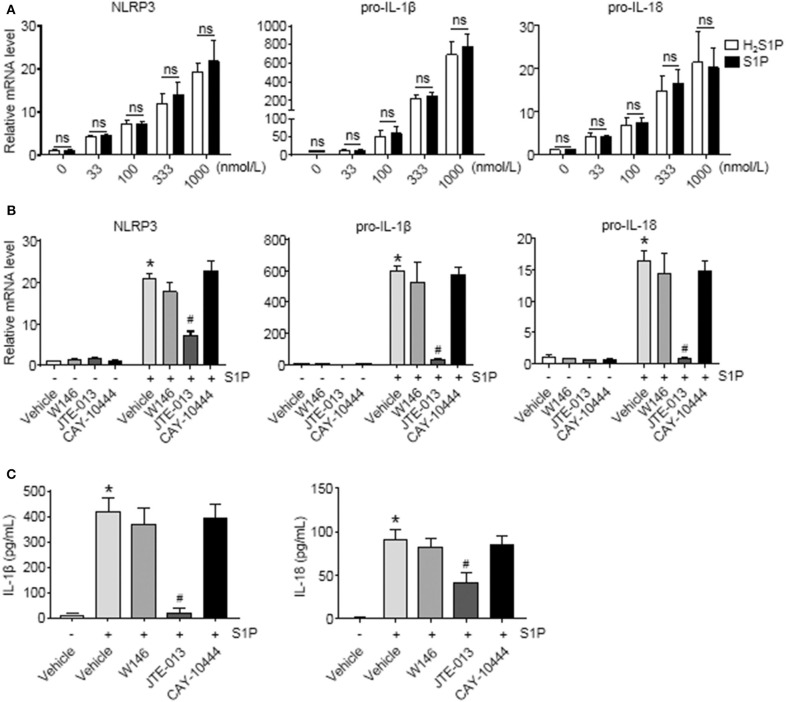
Blockade of S1PR_2_ by JTE-013 reduced NLRP3 inflammasome priming and secretion of mature IL-1β and IL-18 in BMMs. **(A)** The mRNA expression of NLRP3, pro-IL-1β, and pro-IL-18 in serum-starved BMMs treated with the indicated concentrations of S1P or H_2_S1P for 2 h was examined by qRT-PCR. **(B)** serum-starved BMMs were pretreated with S1PR_1_ antagonist W146 (5 μmol/L), S1PR_2_ antagonist JTE-013 (10 μmol/L), and S1PR_3_ antagonist CAY-10444 (10 μmol/L) for 1 h, followed by 1 μmol/L S1P treatment for 2 h. NLRP3, pro-IL-1β, and pro-IL-18 mRNA were examined by qRT-PCR. **(C)** IL-1β and IL-18 in culture supernatants were examined by ELISA. Data are presented as the mean ± SEM. *n* = 6 per group. Student's *t*-test was used in panel **(A)** and one-way ANOVA was used in panels **(B,C)**. *p < 0.05 vs. control, ^#^*p* < 0.05 vs. S1P treatment alone, ns, not significant.

To determine whether S1P induced NLRP3 inflammasome priming via S1PRs, we performed the same experiments using H_2_S1P, a structural analog of S1P that is only able to mediate its effects through S1PRs. Here, we found that the effect of S1P on NLRP3, pro-IL-1β, and pro-IL-18 mRNA expression was completely mimicked by H_2_S1P (Figure 5A). Our previous studies have documented that BMMs abundantly express S1PR_1_, S1PR_2_, and S1PR_3_ (14). Next, we determined which S1PR was implicated in this process. For this purpose, the selective S1PRs antagonists were employed. We found that S1P-induced expression of NLRP3, pro-IL-1β, and pro-IL-18 was blocked by JTE-013 (S1PR_2_ antagonist) (Figure 5B). Pretreatment with W146 (S1PR_1_ antagonist) or CAY-10444 (S1PR_3_ antagonist) did not alter S1P-induced the upregulation of NLRP3, pro-IL-1β, and pro-IL-18 mRNA level (Figure 5B). Moreover, pretreatment with JTE-013 inhibited S1P-induced mature IL-1β and IL-18 secretion, whereas blockade of S1PR_1_ and S1PR_3_ by their selective antagonist had no such effect (Figure 5C).

**Figure 6:**
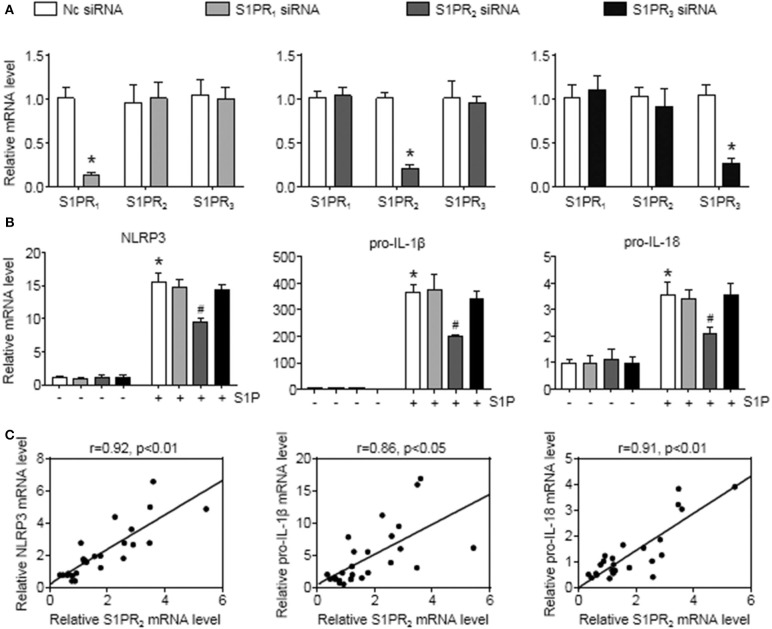
S1PR_2_-siRNA reduced NLRP3, pro-IL-1β, and pro-IL-18 expression in BMMs. **(A)** The efficiency of S1PR_1_, S1PR_2_, and S1PR_3_ knockdown by their siRNAs in serum-starved BMMs. **(B)** Effects of S1PR_1_, S1PR_2_, and S1PR_3_ siRNA on NLRP3, pro-IL-1β, and pro-IL-18 mRNA expression in response to 1 μmol/L S1P for 2 h. **(C)** Mice received an operation of BDL to induce liver injury, then were anesthetized and sacrificed at 1 or 3 days or at 1 or 2 weeks after BDL operation. The relationship between S1PR_2_ and NLRP3, pro-IL-1β, and pro-IL-18 in mouse liver was analyzed by regression analysis. Data are presented as the mean ± SEM. Student's *t*-test was used in panel **(A)** and one-way ANOVA was used in panel **(B)**. *n* = 6 per group. *p < 0.05 vs. control; ^#^*p* < 0.05 vs. S1P treatment alone.

To further investigate this issue, individual S1PRs were knocked down by specific siRNAs. First, we confirmed that S1PR_1_, S1PR_2_, or S1PR_3_ siRNA downregulated each mRNA expression in BMMs and that their mRNA expression was reduced by 85%, 82%, and 76%, respectively (Figure 6A). Second, silencing S1PR_2_ expression abrogated the increase of NLRP3, pro-IL-1β, and pro-IL-18 mRNA expression induced by S1P, whereas siRNA against S1PR_1_ or S1PR_3_ had no effect on that of BMMs (Figure 6B). Furthermore, we noted that the mRNA levels of NLRP3 inflammasome components (NLRP3, pro-IL-1β, and pro-IL-18) exerted positive correlations with the expression of S1PR_2_ in mouse livers (Figure 6C). These results supported that macrophage S1PR_2_ could be an effective therapeutic target for inhibiting NLRP3 inflammasome and alleviating liver inflammation *in vivo*.

### S1PR_2_ siRNA-GeRPs Were Able to Induce Macrophage-Specific Knockdown of S1PR_2_ and Downregulate NLRP3 Inflammasome in Mouse Fibrotic Livers

**Figure 7:**
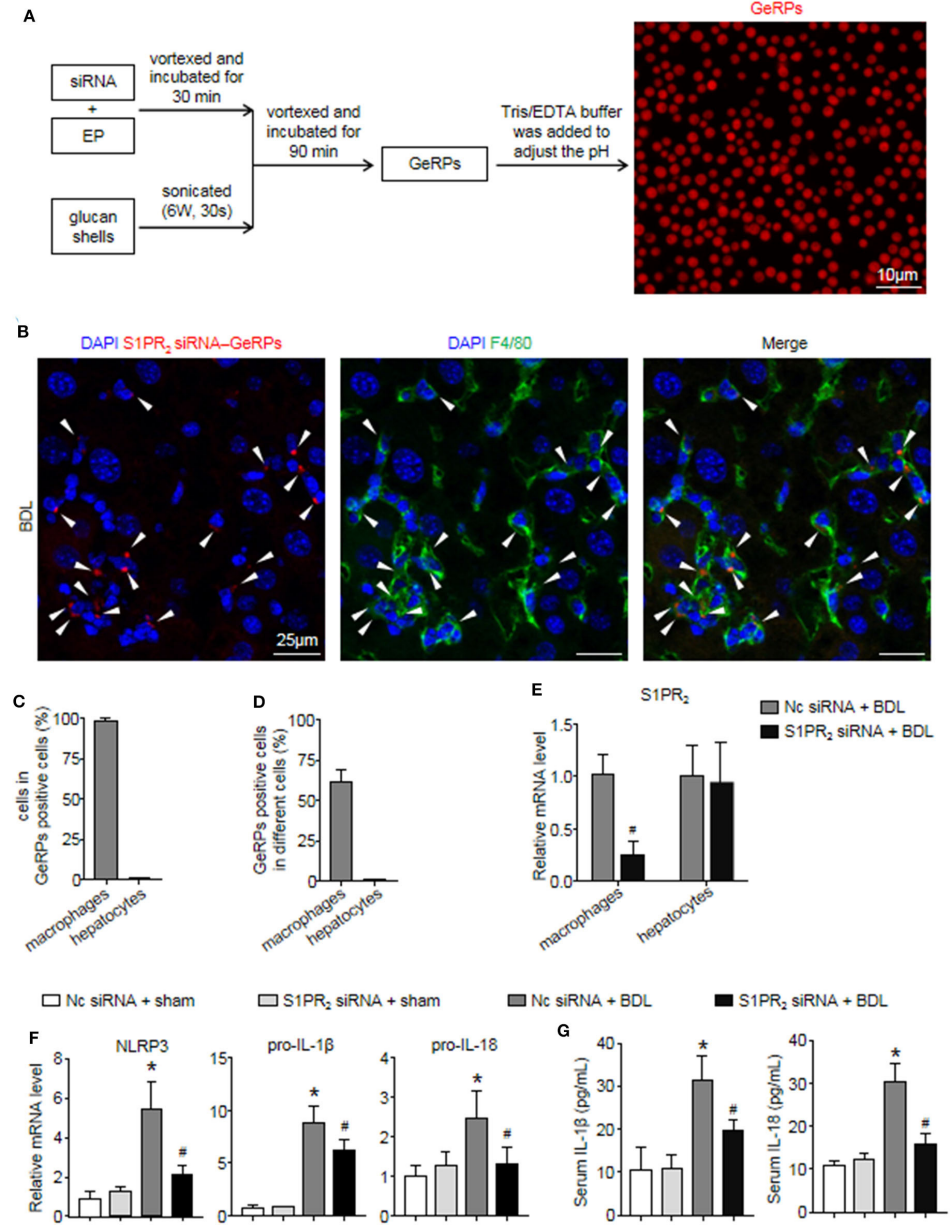
S1PR_2_ siRNA-GeRPs was able to induce macrophage-specific knockdown of S1PR_2_ and downregulate NLRP3 inflammasome in BDL-injured livers. **(A)** Strategy for loading Cy3-labeled glucan shells with siRNA-EP complexes and representative images of GeRPs (red). **(B–D)** Representative images and quantitative analysis of immunofluorescence analysis by confocal microscopy to track the selective uptake of GeRPs (red) in macrophages (F4/80^+^, green) in mouse livers at 2 weeks after BDL operation. **(E)** mRNA expression of S1PR_2_ in macrophages and hepatocytes from mice treated with scrambled or S1PR_2_-siRNA GeRPs. **(F)** mRNA expression of NLRP3, pro-IL-1β, and pro-IL-18 in liver tissues from mice treated with scrambled or S1PR_2_-siRNA GeRPs. **(G)** ELISA analysis of IL-1β and IL-18 levels in serum. Data are presented as the mean ± SEM. *n* = 6 per group. Student's *t*-test was used in panel **(E)** and two-way ANOVA was used in panels **(F,G)**. *p < 0.05 vs. sham group; ^#^*p* < 0.05 vs. BDL-treated alone.

Previous studies have shown that β 1,3-d-glucan–encapsulated the siRNA particle (GeRP) delivery system targets selectivity to macrophages on the basis of their ability to tightly bind and phagocytose such particles (29, 30). To track the formation of GeRPs, glucan shells were labeled with Cy3. GeRPs were made as described (29, 30), and we also studied GeRPs formation by microscopy (Figure 7A). Immunofluorescence staining showed that Cy3-labeled GeRPs, 2–4 μm in diameter, formed at pH 7.4 could be readily detected (Figure 7A).

Then, we injected ICR mice intraperitoneally daily with S1PR_2_ siRNA-GeRPs for 12 days to induce macrophage-specific knockdown of S1PR_2_. Controls received GeRPs containing scrambled siRNA. First, we analyzed the selective uptake for GeRPs in BDL-treated livers. The mean value of 15 randomly selected areas was used to calculate the proportion of macrophages (F4/80^+^) and hepatocytes (large nuclear cells) in Cy3-labeled GeRPs positive cells. Consistent with previous reports (29, 30), GeRPs were significantly enriched in F4/80^+^ macrophages, whereas GeRPs were undetectable in hepatocytes (Figures 7B,C). Besides, most of the macrophages (61.7 ± 5.9%) were able to effectively internalize GeRPs (Figure 7D). S1PR_2_ siRNA-GeRPs treatment resulted in a significant decline of S1PR_2_ mRNA expression in the macrophages of BDL-injured livers, while there was no change of S1PR_2_ mRNA levels in hepatocytes (Figure 7E). Second, we analyzed whether targeting macrophage S1PR_2_ may influence NLRP3 inflammasome priming and activation in cholestatic liver injury. Selective knockdown of macrophage S1PR_2_ by S1PR_2_ siRNA-GeRPs treatment significantly inhibited the expression of NLRP3, pro-IL-1β, and pro-IL-18 in BDL-injured livers compared with scrambled siRNA-GeRPs treatment (Figure 7F). Besides, the secretion of IL-1β and IL-18 were markedly suppressed in mice treated with S1PR_2_ siRNA-GeRPs (Figure 7G). Together, our data supported the notion that targeting macrophage S1PR_2_ attenuated NLRP3 inflammasome-driven inflammation in cholestatic liver injury.

### Selective Knockdown of Macrophage S1PR_2_ Attenuated Liver Inflammation and Fibrosis in BDL-Injured Livers

**Figure 8:**
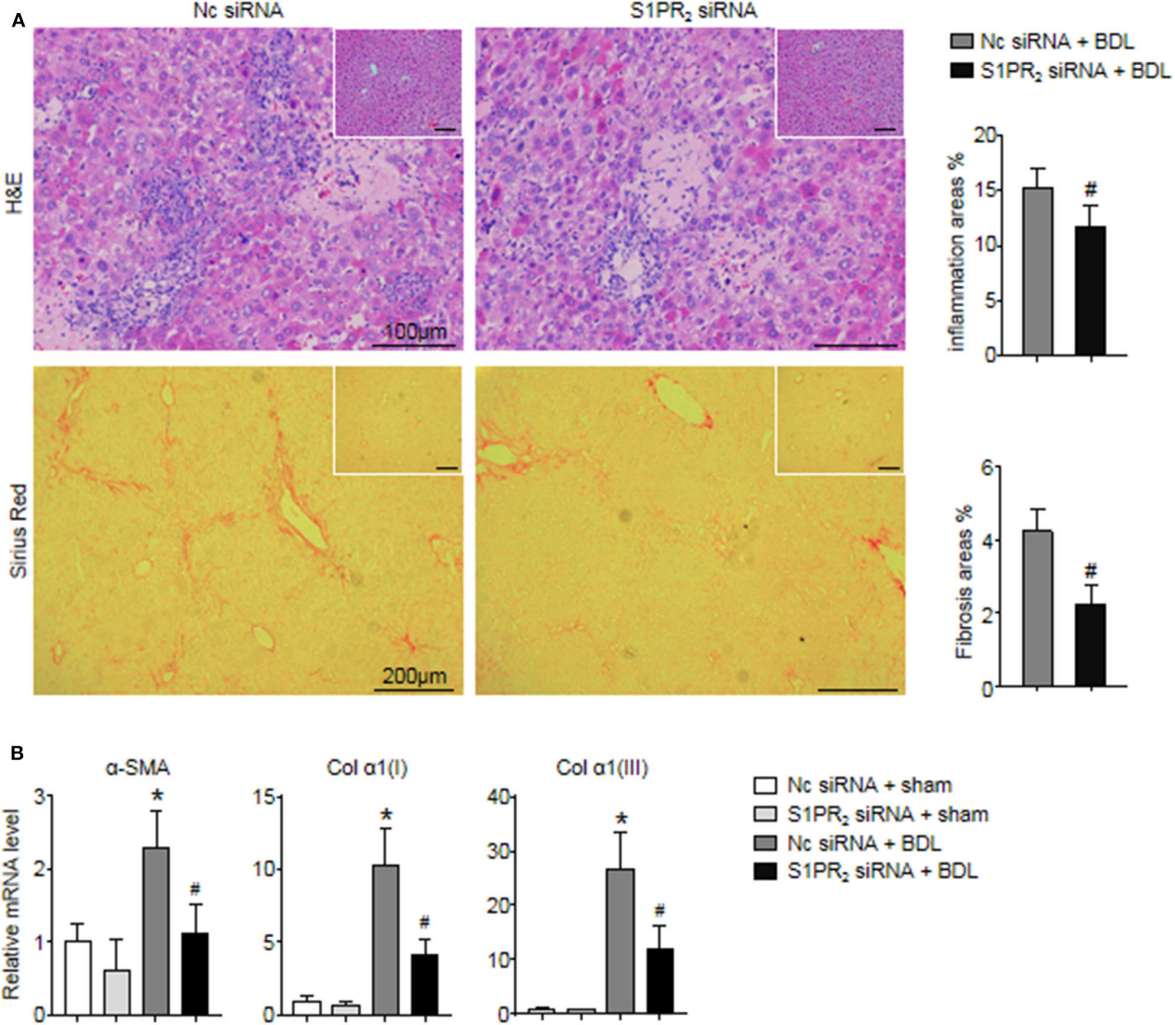
Effects of macrophage-selective siRNA knockdown of S1PR_2_ in BDL-injured livers. **(A)** Representative H&E and Sirius Red staining and quantitative analysis in mouse livers at 2 weeks after BDL operation. Inset: H&E and Sirius Red staining in sham-treated livers. **(B)** Relative mRNA levels of hepatic α-SMA, Col α1(I), and Col α1(III) in mouse livers at 2 weeks after BDL operation. Data are presented as the mean ± SEM. *n* = 6 per group. Student's *t*-test was used in panel **(A)** and two-way ANOVA was used in panel **(B)**. *p < 0.05 vs. sham group; ^#^*p* < 0.05 vs. BDL-treated alone.

In the end, we analyzed whether blockade of macrophage S1PR_2_ signaling may have retarded liver inflammation and fibrogenesis in cholestatic liver injury. First, the inflammation and fibrotic areas were decreased significantly in the injured livers of mice treated with S1PR_2_ siRNA-GeRPs (Figure 8A). Second, BDL mice treated with S1PR_2_ siRNA-GeRPs presented a significant drop in the mRNA expression of fibrosis markers, including α-smooth muscle actin (α -SMA), procollagen α1(I) [Col α1(I)], procollagen α1(III) [Col α1(III)], in BDL-treated livers (Figure 8B). These results validated that treatment with S1PR_2_ siRNA-GeRPs attenuated liver inflammation and fibrogenesis *in vivo*.

## Discussion

Here, our results highlighted the importance of BMMs as key effectors of the NLRP3 function via an overactive S1P/S1PR_2_ system and identified the macrophage S1PR_2_ as a promising molecular and therapeutic target of cholestatic liver injury. Our work provided several new findings as follows: (1) BMMs were the main origin of NLRP3^+^ cells in BDL-injured livers, but not dendritic cells, endothelial cells, T cells, and hepatocytes; (2) S1P induced NLRP3 inflammasome priming and considerable inflammatory cytokine (IL-1β and IL-18) secretion via S1PR_2_ in the absence of another stimulus for NLRP3 inflammasome activation; and (3) selective knockdown of macrophage S1PR_2_ by S1PR_2_ siRNA-GeRPs treatment attenuated liver inflammation and fibrosis via downregulating NLRP3 inflammasome in BDL-injured livers.

The inflammatory effect of NLRP3 inflammasome has been implicated in the development of various chronic liver diseases that lead to fibrosis and cirrhosis (38). Data from several publications suggest that NLRP3 can be expressed both in hepatocytes and hepatic non-parenchymal cells, such as Kupffer cells, dendritic cells, sinusoidal endothelial cells, and stellate cells (5, 31, 39–41). Our current data showed that the majority of NLRP3^+^ cells were F4/80^+^ macrophages (79.5 ± 6.3%), whereas only a small fraction of the NLRP3^+^ cells were other hepatic non-parenchymal cells, including T cells, endothelial cells, and dendritic cells. Notably, BMMs contributed to a significant proportion (92.4 ± 5.6%) of NLRP3^+^ macrophages in BDL-injured livers, while there was no significant change in the expression of NLRP3 in Kupffer cells, supporting that BMMs played pivotal roles in NLRP3 function. Previous studies have provided evidence that NLRP3 exists and is functionally active in hepatocytes (40, 41). In our study, NLRP3 in the livers of BDL-treated mouse colocalize with EGFP^+^ macrophages, but not with hepatocytes. These results supported that BMMs were key effector cells of NLRP3-driven immune responses during cholestatic liver injury.

The inflammatory effect of NLRP3 inflammasome has been implicated in the development of many chronic liver diseases that lead to fibrosis and cirrhosis (38). Here, we demonstrated that S1P specifically induced NLRP3 mRNA expression compared to other inflammasome family members. Although S1P also regulated the expression of AIM2 and NLRC4, the mRNA fold-changes of AIM2 and NLRC4 were much lower than that of NLRP3, implying that S1P is a robust and effective NLRP3 inflammasome inducer. Previous studies show that S1PR_1_, S1PR_2_, and S1PR_3_ are highly expressed in the liver and that the expression levels of S1PR_4_ and S1PR_5_ are too low to be detected (17). Here, we reported that S1P induced considerable IL-1β and IL-18 secretion via S1PR_2_ in BMMs in the absence of another stimulus for NLRP3 inflammasome activation. In another report, LPS/S1PR_1_ signaling participates in NLRP3 expression and IL-1β production in tumor-associated macrophages (42). The reason for such a discrepancy may come from the use of different stimulants or the heterogeneity of macrophage between tumor-associated macrophages and inflammatory macrophages. Furthermore, some pro-inflammatory cytokines, such as TNF-α, also stimulate the gene expression of NLRP3 and pro-IL-1β in macrophages (43, 44), whereas S1P might exert a more powerful effect on NLRP3 inflammasome priming than TNF-α (43, 44).

S1P/S1PRs signaling has been indicated to regulate immune response in various inflammatory diseases (10, 14, 25). In this study, we noted that S1P was a powerful pro-inflammatory molecular in both promoting NLRP3 inflammasome priming and activation and regulating M1 phenotype polarization of BMMs. While a previous study has shown that S1P reduces LPS-induced inflammatory gene expression and could regulate the inflammatory phenotype of LPS-stimulated mouse macrophages (45), the reason for such a discrepancy might come from the difference between C57BL/6 mice and ICR mice. Our previous results have documented that BMMs from ICR mice abundantly expressed S1PR_1_, S1PR_2_, and S1PR_3_, whereas Hughes JE et al. found no expression of S1PR_3_ in BMMs from C57BL/6 mice (15, 45). Interestingly, Hughes JE et al. noted that S1P induced a significant increase of TNF-α, and had no effects on Arginase-1 expression, which were similar to our results (45). Further studies will be needed to illustrate the role of S1P/S1PRs signaling in regulating phenotype polarization of BMMs in inflammatory diseases.

In consideration of the importance of S1PRs for the treatment of multiple inflammatory diseases, drugs targeting S1PRs have been developed. Fingolimod, the first S1PRs modulator, has been approved by the United States Food and Drug Administration to treat relapsing multiple sclerosis (27, 46). Owing to the ubiquitous and overlapping expression of S1PRs in various kinds of cells, fingolimod treatment can cause undesirable side effects as a result of its interaction with other S1PRs subtypes (27). In addition, S1PRs is also involved in bile acid signaling in the liver. Conjugated bile acids increase nuclear S1P and induce genes encoding enzymes and transporters involved in glucose, lipid, and sterol metabolism in hepatocytes via S1PR_2_ (47, 48). Thus, the traditional delivery method, systemic administration, can lead to systemic side effects and low bioavailability when high doses are required at a localized area (28, 49). Recently, nucleic acid therapeutics, such as sequence-specific gene silencing by siRNA, are also being explored as novel therapeutic strategies for a variety of diseases (30, 49, 50). Development of a strategy to efficiently deliver pharmacological inhibitors or siRNA into specific cells, such as macrophages, is expected to be a more effective therapy for the treatment of liver diseases (30, 51).

In this study, one striking finding was that selective knockdown of the macrophage S1PR_2_ replicated the effect of JTE-013 in remarkable attenuation of NLRP3 inflammasome priming and activation during cholestatic liver injury (data not shown). Increasingly, conjugated bile acids have also been implicated in various inflammatory diseases by activating specific nuclear receptors and G protein-coupled receptors (GPCRs), such as S1PR_2_ (52, 53). In a recent study, researchers found that blockage of S1PR_2_ substantially reduced mature IL-1β production and alleviated colonic inflammation induced by conjugated bile acids (53). Our previous studies have documented that a significant proportion (48.2 ± 3.5%) of hepatic non-parenchymal cells are BMMs, supporting the important role of macrophages in BDL-induced liver injury (34). Selective knockdown of macrophage S1PR_2_ also attenuated the hepatic inflammation and fibrosis in cholestatic liver injury, as compared to scrambled siRNA-treated controls. Consistent with previous reports, only F4/80^+^ macrophages, which have strong phagocytic activity, can effectively internalize GeRPs (29, 30). Moreover, GeRPs are undetectable in peripheral blood monocytes, while GeRPs are enriched ~10-fold in CD68^+^ macrophages compared to CD3^+^ lymphocytes (29, 30). Thus, the GeRP delivery system is capable of targeting selectivity to macrophages and inducing macrophage-specific gene silence. As NLRP3 mainly expressed and functioned in macrophages via S1P/S1PR_2_ signaling, targeting macrophage S1PR_2_ with S1PR_2_ siRNA-GeRPs was able to effectively inactivate macrophage NLRP3 inflammasome and avoid the potential risks of undesirable side effects. In summary, our results demonstrated that GeRPs-mediated gene silence of macrophage S1PR_2_ attenuated liver inflammation and fibrogenesis triggered by NLRP3 inflammasome, which provided an example for the treatment of chronic liver injury in the near future.

## Data Availability Statement

The datasets generated for this study are available on request to the corresponding author.

## Ethics Statement

The animal study was reviewed and approved by the Ethics Committee of Capital Medical University. The study was conducted in accordance with the Declaration of Helsinki, and the protocol was approved by the Ethics Committee of Beijing Shijitan Hospital, Capital Medical University, Beijing, China (Project identification code: 2018EC-1). The patients/participants provided their written informed consent to participate in this study.

## Author Contributions

LL conceived and designed the study. LH designed the research studies, conducted experiments, acquired data, and analyzed data. LH, LeY, NC, and LL drafted the manuscript. XZhao, XZhou, and LinY conducted experiments, acquired data, and provided reagents. CD and FL provided human specimens and collected data. All authors contributed to the article and approved the submitted version.

## Conflict of Interest

The authors declare that the research was conducted in the absence of any commercial or financial relationships that could be construed as a potential conflict of interest.

## Abbreviations

NLR: nod-like receptor
NLRP3, NLR family: pyrin domain containing 3
AIM2: absent in melanoma 2
NLRC4, NLR family: CARD domain containing 4
Nlrp1b, NLR family: pyrin domain containing 1B
ASC: apoptosis-associated speck-like protein containing a CARD
IL: interleukin
BDL: bile duct ligation
MCDHF diet: methionine-choline-deficient and high-fat diet
OO: olive oil
CCl4: carbon tetrachloride
BM: bone marrow
BMM: bone marrow-derived monocyte/macrophage
S1P: Sphingosine-1-phosphate
S1PR: S1P Receptor
SphK1: sphingosine kinase 1
LPS: Lipopolysaccharide
ATP: adenosine 5′-triphosphate disodium salt
GeRPs, β1: 3-d-glucan–encapsulated siRNA particles.
