# Supplementary material for: Macrophage Sphingosine 1-Phosphate Receptor 2 Blockade Attenuates Liver Inflammation and Fibrogenesis Triggered by NLRP3 Inflammasome
Source: Front Immunol. 2020 Jun 26;11:1149. doi: 10.3389/fimmu.2020.01149 (PMC7333785; doi:10.3389/fimmu.2020.01149)
Supplement: Supplementary file 1 [file Image_1.pdf]

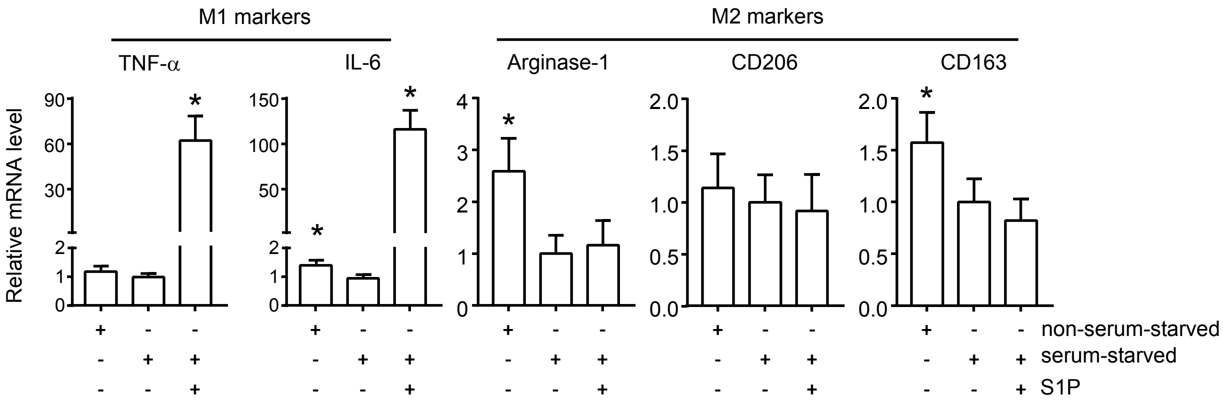

Supplementary Figure 1. S1P induces BMMs polarized toward M1 type *in vitro.* The mRNA expression of M1 markers (TNF-α and IL-6), and M2 markers (Arginase-1, CD206 and CD163) in BMMs treated with non-serum-starved, serum-starved or 1 μmol/L S1P for 2 hours after serum-starvation. Data are presented as mean ± SEM; n=6 per group; * $P<0.05$ compared with serum-starved group.
